# AI-assisted diagnosis of anemia through peripheral smear image analysis: A cross-validation study

**DOI:** 10.6026/973206300213668

**Published:** 2025-10-31

**Authors:** Ashita Nain, Sangeeta Gupta, Sylvester Noeldoss Lazarus, Kawalinder Kaur Girgla, Parth Jani, Amrit Podder, Sreemoyee Dutta, Ravi Babu Surisetti

**Affiliations:** 1 Department of Physiology, Lala Lajpat Rai Memorial Medical College, Meerut Uttar Pradesh, India; 2 Department of Physiology, AIIMS Gorakhpur, Uttar Pradesh, India; 3 Department of Pathology, American University of Barbados, Wildey, Barbados (Caricom); 4 Department of Physiology, SGRD Institute of Medical Sciences and Research, Sri Amritsar, Punjab, India; 5 Department of General Medicine, All India Institute of Medical Sciences, Rajkot, Gujarat, India; 6 Department of Physiology, Teerthanker Mahaveer Medical College & Research Centre, Teerthanker Mahaveer University, Moradabad, Uttar Pradesh, India; 7 Department of Periodontology, Kalinga Institute of Dental Sciences, Bhubaneswar, Odisha, India; 8 Department of Zoology, Trans, Disciplinary Research Hub, Andhra University, Visakhapatnam Andhra Pradesh, India

**Keywords:** Anemia diagnosis, deep learning, peripheral blood smear, semi-supervised learning, red blood cells, medical image analysis

## Abstract

A deep semi-supervised learning model for automating anemia detection and classification from peripheral blood smear images is of interest. A convolutional neural network was trained on 3,200 images, with only 25% annotated by expert hematologists. The model achieved a classification accuracy of 93.4% and F1-scores above 90% for key anemia subtypes, demonstrating strong agreement with expert diagnoses (κ = 0.89). It significantly reduced diagnostic time and performed well in detecting microcytic and sickle cell anemia. This AI-based framework shows great potential for accurate anemia diagnosis, especially in resource-limited settings.

## Background:

Anemia, a prevalent hematology disorder characterized by a lack of red blood cells (RBCs) or hemoglobin, afflicts billions of individuals and is a major global health burden [1]. Traditional methods like complete blood count (CBC) and manual peripheral blood smear examination are still standard but are plagued by subjectivity and inter-observer variation [2]. Morphological RBC examination in peripheral smears is a critical diagnostic tool for anemia variants, but manual examination is time-consuming and effort-scarce in terms of expertise [3]. Recent advances in artificial intelligence (AI) and particularly deep learning have facilitated the exploration of new fields for automating and enhancing diagnostic precision [4]. Several research studies have established the capability of AI-based systems in detecting morphological disorders in RBCs with good accuracy, such as sickle cell anemia and malaria anemia [5]. Furthermore, the application of semi-supervised learning models has proven to be potential in overcoming the dependency on large annotated datasets, significantly lowering the annotation cost while maintaining strong performance [6]. Interestingly, deep semi-supervised models have been successfully applied to peripheral smear examination for tracking recovery from anemia, pointing to the utility of such techniques in longitudinal clinical settings [7]. In addition, the presence of high-quality curated datasets like the Ane RBC benchmark has allowed the training and validation of AI models tailored for anemia diagnosis based on RBC images [8, 9]. In this paper, we propose an artificial intelligence-based diagnostic system for anemia detection based on peripheral blood smear image analysis. Using cross-validation techniques, we assess the model's accuracy in anemic trait detection and examine its deployability in a clinical setting. Therefore, it is of interest to bridge the gap between visual microscopy and computer-aided diagnosis, increasing efficiency, accuracy and accessibility in anemia detection.

## Methodology:

This research utilized a cross-sectional study combining AI-based image analysis with clinical correlation. Peripheral blood smear specimens were obtained from a heterogeneous population of patients with suspected anemia at a tertiary medical center. Blood samples were Wright-Giemsa stained according to standard practice and scanned using a high-resolution microscope scanner at 100x magnification. A total of 3,200 images were obtained and included normal and a range of anemic morphologies, such as microcytic, macrocytic, hypochromic and sickle-shaped RBCs. Images were anonymized and confirmed by experienced hematopathologists to guarantee diagnostic accuracy and reproducibility. Senior hematologists labeled a random sample of 800 images, identifying salient morphological features relevant to anemia classification, such as anisocytosis, poikilocytosis and hemoglobin content variation. Labelled images served as ground truth for supervised learning, while the remaining 2,400 images were utilized during semi-supervised learning. Inter-observer reliability was determined using Cohen's kappa coefficient, which provided a measure of 0.87, thus indicating high reliability in labeling. A deep semi-supervised convolutional neural network (CNN) was developed, inspired by previously established designs in hematological image processing. The model consisted of a sequence of convolutional layers for feature learning, with classification being performed using dense layers. The pseudo-label method was applied to the unlabeled set for enhancing training efficiency without the cumbersome addition of annotation costs. Data augmentation techniques like rotation, flipping and color normalization were applied to reduce over fitting risk and enhance generalizability. For comparison of model performance, five-fold cross-validation was used to ensure that observations from the same patient were not split between the train and validation set. Model predictions were compared against expert diagnoses using precision, recall, F1-score and accuracy. The model performed with a mean classification accuracy of 93.4%, F1-score of 0.91 for the detection of microcytic anemia and 0.89 for macrocytic types. Statistical calculations were carried out using Python (version 3.10) and the SciPy library. Human expert and AI model diagnostic performance differences were compared via paired t-tests at an alpha level of p < 0.05. All notable performance measures were reported using 95% confidence intervals to ascertain robustness of findings.

## Results:

The AI-aided model's performance was tested on different anemia subtypes based on a corpus of 3,200 peripheral blood smear images. The findings proved the high diagnostic accuracy of the model in identifying and classifying types of anemia with strong agreement with expert hematologist annotations. Here, we provide the results in the following order of classification accuracy, subtype-specific performance and comparative investigation of expert diagnoses. The deep semi-supervised model attained high overall diagnostic accuracy. Its average precision, recall and F1-score for all anemia classes were 92.1%, 91.6% and 91.8%, respectively. The confusion matrix analysis showed a good capability to distinguish between normal and abnormal red cell morphologies with very few misclassifications (Table 1 - see PDF). Figure 1 (see PDF) illustrates the model's ability to discriminate between anemia types, with all curves showing high area under the curve (AUC) values, especially for microcytic and sickle cell anemia, indicating strong classification performance. Figure 2 (see PDF) visually highlights the distribution of true vs. predicted labels, with the highest concentration along the diagonal, confirming the model's accuracy in classifying most smear images correctly. These findings all together validate the performance of the AI-supported diagnostic platform in classifying anemia types correctly using peripheral blood smear examination with performance measures on par with or higher than human experts in some categories. Subtype classification indicated subtle differences in performance. The model was highest in classifying microcytic anemia with an F1-score of 94.2%. Macrocytic anemia and normocytic hypochromic anemia were also high in classification scores, albeit somewhat lower, potentially resulting from shared morphological characteristics (Table 2 - see PDF). When comparing the expert hematologist ratings with model predictions, the rate of agreement was 94.1%, as quantified by Cohen's kappa (κ = 0.89), indicating outstanding consistency. Time efficiency was also notably enhanced, with the model taking 3.2 seconds on average per slide analysis, in contrast to manual examination at 5-7 minutes.

## Discussion:

The decrease in cost of annotation indicated in our research is also aligned with the efficiency that comes in Semi-HIC, a model used in histopathological image classification as shown by Su *et al.* (2021) [10], in evidence of cross-domain usability of such models. The findings of this research establish that deep semi-supervised learning-based AI-aided diagnosis is a robust, effective and scalable method for anemia detection via peripheral smear examination. The model attained high classification accuracy on various anemia subtypes, with exceptionally good performance in detecting microcytic and sickle cell anemia, coming close to expert-level diagnoses. These results are consistent with previous research in red blood cell (RBC) image classification, e.g., Xu M *et al.* (2017) [11], who proposed a CNN-based model for sickle cell anemia identification with comparable high performance, confirming the power of deep learning approaches in hematological imaging. The semi-supervised learning method utilized herein has a significant benefit in de-burdening annotation by only having a subset of the dataset labeled by an expert to enable high model generalizability. This is similar to what has been reported in other applications, e.g., mammography and abdominal organ segmentation as discussed by Cho & Lee (2024) [12], where semi-supervised techniques supported high diagnostic accuracy with low amounts of labeled data. Relative to standard diagnostic processes, the automated system not only cut down the time spent per analysis of a slide from multiple minutes to a few seconds but also retained high interpretability and reliability. This effectiveness is especially useful in high-volume or low-resource clinical environments, consistent with findings by Lee *et al.* (2024) [14], who achieved real-time performance gains in interpreting coronary angiograms with semi-supervised models. The model's capacity to discriminate between fine morphological variations within RBCs i.e., deformability and shape abnormalities also improves on earlier observations by Lamoureux *et al.* (2022) [13], who used deep learning for the measurement of RBC deformability in microfluidic assays. Inclusion of these morphological indicators by the current work adds diagnostic detail in anemia categorization and facilitates the use of such tools in standard hematological testing. Furthermore, the identification of cases of malaria anemia-captured in the model's ability to detect sickle-like or fragmented RBCs can be of clinical significance in endemic areas. This converges with the findings of Crider *et al.* (2022) [15], who highlighted the difficulty of diagnosing anemia in malaria-endemic areas, where coinfections and micronutrient deficiencies can complicate clinical assessment.

## Conclusion:

We show that a deep semi-supervised learning model can accurately diagnose anemia from peripheral blood smear images with minimal annotation requirements. The model matched expert performance, enhancing diagnostic efficiency and speed. Thus, we show the potential of AI-assisted systems to improve hematological diagnostics, particularly in resource-limited settings.
